# Structural and Dynamical Characteristics of Short-Chain Branched Ring Polymer Melts at Interface under Shear Flow

**DOI:** 10.3390/polym12123068

**Published:** 2020-12-21

**Authors:** Seung Heum Jeong, Soowon Cho, Tae Yong Ha, Eun Jung Roh, Chunggi Baig

**Affiliations:** 1 Ulsan National Institute of Science and Technology (UNIST), School of Energy and Chemical Engineering, UNIST-gil 50, Eonyang-eup, Ulju-gun, Ulsan 689-798, Korea; shjung252@unist.ac.kr (S.H.J.); tnqhd123@unist.ac.kr (S.C.); gkxodyd0709@unist.ac.kr (T.Y.H.); 2 KOLON Advanced Research Cluster, KOLON One & Only Tower, 110, Magokdong-ro, Gangseo-gu, Seoul 07793, Korea; eunjung_roh@kolon.com

**Keywords:** molecular dynamics, confined system, ring polymer, short-chain branches

## Abstract

We present a detailed analysis of the interfacial chain structure and dynamics of confined polymer melt systems under shear over a wide range of flow strengths using atomistic nonequilibrium molecular dynamics simulations, paying particular attention to the rheological influence of the closed-loop ring geometry and short-chain branching. We analyzed the interfacial slip, characteristic molecular mechanisms, and deformed chain conformations in response to the applied flow for linear, ring, short-chain branched (SCB) linear, and SCB ring polyethylene melts. The ring topology generally enlarges the interfacial chain dimension along the neutral direction, enhancing the dynamic friction of interfacial chains moving against the wall in the flow direction. This leads to a relatively smaller degree of slip ($d_{s}$) for the ring-shaped polymers compared with their linear analogues. Furthermore, short-chain branching generally resulted in more compact and less deformed chain structures via the intrinsically fast random motions of the short branches. The short branches tend to be oriented more perpendicular (i.e., aligned in the neutral direction) than parallel to the backbone, which is mostly aligned in the flow direction, thereby enhancing the dynamic wall friction of the moving interfacial chains toward the flow direction. These features afford a relatively lower $d_{s}$ and less variation in $d_{s}$ in the weak-to-intermediate flow regimes. Accordingly, the interfacial SCB ring system displayed the lowest $d_{s}$ among the studied polymer systems throughout these regimes owing to the synergetic effects of ring geometry and short-chain branching. On the contrary, the structural disturbance exerted by the highly mobile short branches promotes the detachment of interfacial chains from the wall at strong flow fields, which results in steeper increasing behavior of the interfacial slip for the SCB polymers in the strong flow regime compared to the pure linear and ring polymers.

## 1. Introduction

Interfacial polymeric liquids in confined systems exhibit a variety of distinctive properties and phenomena compared to ordinary bulk polymeric liquids, such as solid-like behavior, high viscosity, oscillatory solvation force, and extrusion instability [1,2,3,4,5,6,7,8]. Numerous experimental, computational, and theoretical studies have been conducted for a variety of confined polymeric systems with respect to the wall roughness, degree of wettability, polymer molecular weight, and degree of confinement [9,10,11,12,13,14,15,16,17,18,19]. An important physical behavior that occurs in confined polymer systems is the interfacial slip of polymer chains at the boundary walls, which is considered one of the major physical origins of the melt instability phenomena of polymer extrudates during practical extrusion processes. The slip behavior essentially represents the interfacial dynamic interactions between the polymer chains and the wall surface [6,7,8,20,21,22].

Recently, in an effort to elucidate the fundamental molecular characteristics underlying interfacial polymer slip, several nonequilibrium molecular dynamics (NEMD) simulations have been conducted for various confined polymer melt systems [23,24,25,26]. For instance, we previously quantified the degree of slip for confined linear polyethylene (PE) melts under steady shear flow with respect to the flow strength and revealed the underlying molecular mechanisms in conjunction with the interfacial chain configurations [23]. We further examined the interfacial slip for short-chain branched (SCB) linear PE melts and reported the slip behavior of the SCB polymer to be quite distinct from that of the corresponding linear polymer, which was ascribed to the generally more compact and less deformed chain structures of the SCB polymer in response to the applied flow via the intrinsically fast random motions of short branches [25,27,28]. In addition, ring polymers, with their closed-loop molecular geometry, are known to exhibit a lower degree of chain stretching, weaker shear thinning, reduced interfacial slip, and hydrodynamic inflation toward the neutral direction under shear flow [26,29,30,31,32].

In this work, we further extended the previous studies to investigate the combined (or synergetic) influence of the ring topology and short-chain branching on the interfacial rheology. In particular, we conducted a detailed analysis of the general structural and dynamical characteristics of interfacial chains for confined SCB ring melt systems under shear flow using atomistic NEMD simulations. The results were directly compared to those for the corresponding linear, ring, and SCB linear systems.

## 2. Systems Studied and Simulation Methods

In this study, we simulated four different monodisperse PE melts: C_128_H_258_ linear PE (denoted “Linear”), C_128_H_256_ ring PE (denoted “Ring”), C_178_H_358_ SCB linear PE (denoted “SCB_L”), and C_178_H_356_ SCB ring PE (denoted “SCB_R”). The backbone length was maintained at 128 carbon atoms for all four systems. Each SCB ring or linear molecule contained 10 short branches uniformly distributed along the backbone, with each branch containing five carbon atoms. Atomistic canonical NEMD simulations were performed a constant temperature of *T* = 450 K for all of the systems with densities of ρ = 0.7767, 0.7895, 0.7821, and 0.7835 g/cm^3^ for the linear, ring, SCB linear, and SCB ring PE melts, respectively (corresponding to a pressure of *p* = 1 atm for each confined system).

Each system was subjected to a simple shear flow imposed by moving the top wall at a constant velocity $V_{w}$ in the flow (*x*-)direction while keeping the bottom wall fixed. The basic simulation box dimensions confined by rigid simple cubic lattice walls were set to (68.30 Å, 66.54 Å, 68.30 Å) (containing 81 molecules) for the linear PE melt, (68.30 Å, 65.45 Å, 68.30 Å) (containing 81 molecules) for the ring PE melt, (68.30 Å, 61.25 Å, 68.30 Å) (containing 54 molecules) for the SCB linear PE melt, and (88.12 Å, 76.05 Å, 94.56 Å) (containing 120 molecules) for the SCB ring PE melt, expressed as (*x*, *y*, *z*) where *x*, *y*, and *z* represent the flow, velocity gradient, and neutral directions, respectively. The box dimensions in the *y*- and *z*-directions of the systems were set as more than three times the chain radius of gyration *R*_g_. The simulation boxes were enlarged in the flow direction by duplicating them by one, two, and three times to prevent system-size effects under strong flow fields. The simulation systems were confined by rigid simple cubic lattice walls composed of 676, 1352, and 2028 atoms for each of the linear, ring, and SCB linear PE melts and 1224, 2448, and 3672 atoms for the SCB ring PE melts. Each wall atom was kept fixed in its lattice site during the simulations. 

The atomistic NEMD simulations of the PE melts under shear flow were executed with the *p*-SLLOD algorithm, implemented by the Nosé–Hoover thermostat [33,34,35,36]. The well-known transferable potentials for phase equilibria (TraPPE) united-atom model was adopted for all of the systems [37]. The set of evolution equations was numerically integrated using the reversible reference system propagator algorithm (*r*-RESPA) with two different time scales in an MD step: 0.48 fs for the three bonded (bond-stretching, bond-bending, and bond-torsional) interactions and 2.39 fs for the nonbonded inter- and intramolecular Lennard–Jones (LJ) interactions, the thermostat, and the flow field (see the Supporting Information of Ref. 39 for the detailed *r*-RESPA formula) [38,39].

The lattice parameter of the simple cubic wall corresponded to the LJ size parameter of the wall atoms $\sigma_{w}$ = 1.33 $\sigma_{{}_{CH_{2}}}$ for all systems [23]. The LJ energy parameter of the wall atoms was set to ε_w_/*k_B_* = 939 K, which is comparable to that of a mica surface (ca. 200–400 mJ/m^2^) [19]. All of the systems were subjected to steady shear flow over a wide range of shear rates $\dot{\gamma }$, $0\leq {\dot{\gamma }}^{*}\equiv \dot{\gamma }\sqrt{m\sigma^{2}/\varepsilon }\leq 0.2$, where *m* (=14 *g*/mol), σ (=3.95 Å), and ε (ε*/k**_B_***= 46 K where *k_B_* is Boltzmann’s constant) denote the mass, size, and energy parameter for the CH_2_ units, respectively. The longest characteristic relaxation time τ_R_ of the system was as follows: $\tau_{R}$ = 20.7 ± 4.5 ns, 2.55 ± 0.4 ns, 35.5 ± 7.2 ns, and 9.4 ± 1.0 ns for the simulated linear, ring, SCB linear, and SCB ring PE melts, respectively. The $\tau_{R}$ was estimated from the integral below the stretched exponential curve describing the decay of the time autocorrelation function of the unit chain end-to-end vector for linear polymers and the unit ring diameter vector for ring polymers [29]. To obtain statistically accurate results, the simulation for each system was executed for a sufficiently long time (e.g., more than 5 times the longest relaxation time of the system). Additional details of the potential model and simulation method are described in the Supplementary Materials.

## 3. Results and Discussion

Following the previous studies [23,24,25,26], here we analyzed the degree of slip (*d*_s_) for the confined polymeric system defined as $d_{s}$ = 1 − ${\dot{\gamma }}_{real}$/${\dot{\gamma }}_{ideal}$ = $V_{s}/V_{w}$, where $V_{s}$ is the total slip velocity occurring at the top and bottom walls and $V_{w}$ is the applied velocity of the moving top wall toward the flow direction in shear flow. Whereas the ideal (nominal) shear rate ${\dot{\gamma }}_{ideal}$ = $V_{w}/H$ is based on the assumption of the no-slip boundary condition with *H* being the box dimension in the velocity gradient (*y*-)direction, the real shear rate ${\dot{\gamma }}_{real}$ = $\left(V_{w}-V_{s}\right)/H$ accounts for a finite slip at the boundary walls. Practically, we evaluated the value of ${\dot{\gamma }}_{real}$ for the *d*_s_ of the confined systems as follows: the *y*-dimension of the simulation box (i.e., *H*) was divided into several bins with a constant interval, and the streaming velocity in the flow direction for each bin was calculated by averaging the *x* component of velocity for all atoms belonging to that bin. Then, the final streaming velocity was obtained by applying a fifth-order polynomial fitting to the velocity data measured in each MD step and averaging the resulting velocity profile over a sufficiently long system trajectory (see the Supporting Information of [23] for additional details). ${\dot{\gamma }}_{real}$ was then calculated by applying linear regression to the average streaming velocity data along the velocity gradient direction. We also note that the degree of slip is directly related to the standard slip length ($L_{s}$) by $d_{s}^{-1}=1+{\tilde{L}}_{s}^{-1}$ with ${\tilde{L}}_{s}^{}\equiv L_{s}/H$.

**Figure 1:**
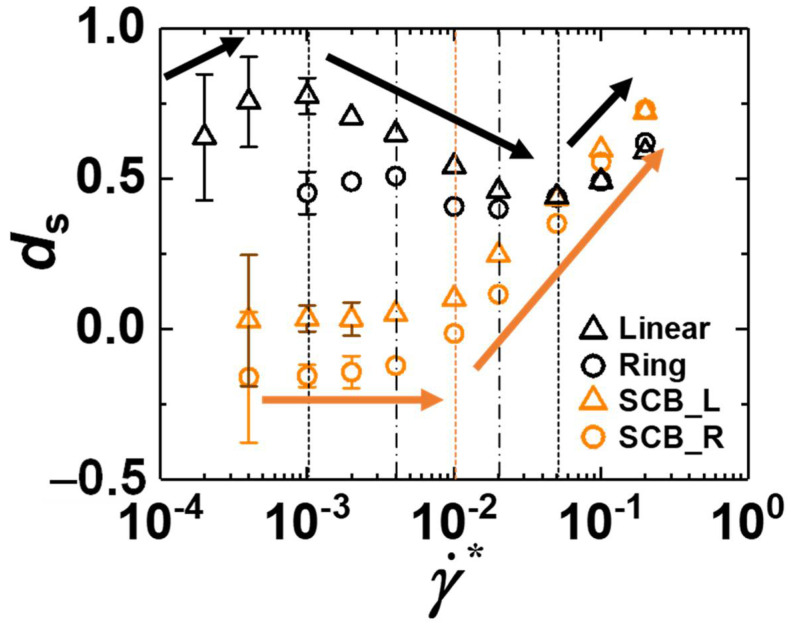
Degree of slip ($d_{s}$) as a function of the reduced shear rate ${\dot{\gamma }}^{*}\equiv \dot{\gamma }\sqrt{m\sigma^{2}/\varepsilon }$ for the simulated C_128_H_258_ linear (Linear; black triangles), C_128_H_256_ ring (Ring; black circles), C_178_H_358_ SCB linear (SCB_L; orange triangles), and C_178_H_356_ SCB ring (SCB_R; orange circles) PE melt systems. The vertical black dashed lines (Linear), black dash-dotted lines (Ring), and orange dashed line (SCB_L and SCB_R) separate the characteristic flow regimes with respect to *d*_s_ for each system. It is noted that while the linear and ring polymers exhibit three distinct characteristic *d*_s_ regimes (increasing, decreasing, and increasing) as a function of shear rate, the SCB systems show almost constant behavior of $d_{s}$ in the weak and intermediate flow regimes and increasing behavior of *d*_s_ in the strong flow regime. The error bars are smaller than the size of the symbols unless otherwise indicated.

Figure 1 presents the variation of the degree of slip for the simulated linear C_128_H_258_, ring C_128_H_256_, SCB linear C_178_H_358_, and SCB ring C_178_H_356_ PE melts with respect to the applied shear rate over a wide range of flow strengths for the confined systems. The slip behavior of $d_{s}$ for similar linear, ring, and SCB linear PE melts under shear flow was previously investigated in conjunction with the underlying molecular mechanisms [23,25,26]. To help interpret the general behavior of interfacial slip for the SCB ring melts in view of the combined rheological influence of the ring geometry and short-chain branching, here we analyzed the degree of slip and characteristic molecular mechanisms for the SCB ring polymer via direct comparison with those for the corresponding linear, ring, and SCB linear polymers. First, we note in Figure 1 that the pure linear and ring melts exhibit three distinct characteristic $d_{s}$ regimes with respect to the shear rate, i.e., an increasing, decreasing, and increasing behavior of $d_{s}$ in the weak, intermediate, and strong flow regimes, respectively. In contrast, the SCB linear and SCB ring melts display almost constant behavior of $d_{s}$ in the weak-to-intermediate flow regimes, followed by a rapid increase of $d_{s}$ in the strong flow regime. To understand these distinctive slip behaviors of $d_{s}$ for each system, it is essential to examine the fundamental molecular mechanisms of polymer chains at interfaces as a function of the applied shear rate by considering the dynamical influences of the external flow field and polymer–wall interactions [23,24,25,26].

**Figure 2:**
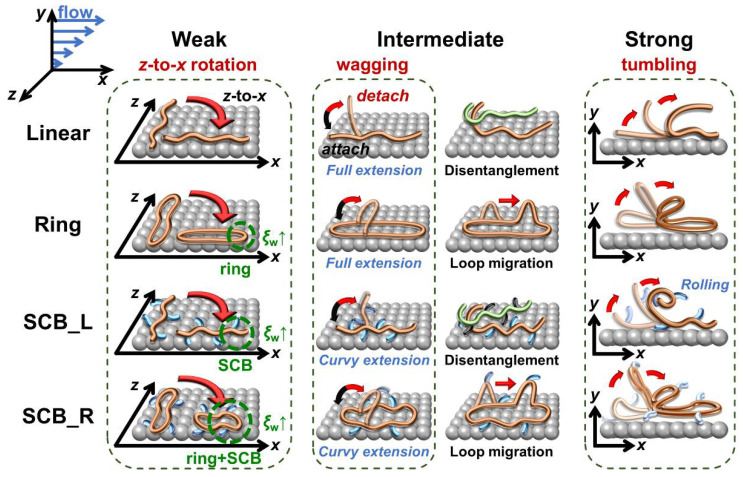
Schematic description of the characteristic molecular mechanisms for the interfacial linear, ring, SCB linear, and SCB ring polymers in the three representative (weak, intermediate, and strong) flow regimes. These mechanisms underlie the general behavior of the interfacial slip ($d_{s}$) for each system. $\xi_{w}$ denotes the polymer-wall friction coefficient.

Figure 2 depicts the characteristic molecular mechanisms for each polymer system in the three representative (weak, intermediate, and strong) flow regimes. To systematically understand the influence of the ring and short-chain branches on the interfacial chain dynamics, we illustrate the mechanisms of the linear polymer as basis.

In the weak flow regime, the applied shear force induces the interfacial linear chains to undergo *z*-to-*x* chain rotation while residing in the *xz*-plane, and the degree of chain alignment in the flow direction increases as the shear rate increases. This effectively reduces the dynamic friction of polymer chains moving against the wall in the flow direction, thereby increasing the degree of slip [23]. The ring polymer also exhibits a similar in-plane *z*-to-*x* rotation, enhancing the interfacial slip. However, in comparison to the linear polymer, the ring polymer has a relatively larger chain dimension in the neutral (*z*-)direction owing to its intrinsic closed-loop molecular geometry, which promotes the dynamic friction of ring chains against the wall. This leads to a relatively smaller $d_{s}$ value and less pronounced increase in $d_{s}$ for the ring polymer in the weak flow regime in comparison to the linear analogue, as observed in Figure 1.

Looking into the effect of short-chain branching, the SCB linear polymer similarly exhibits *z*-to-*x* chain rotation, thus reducing the overall interfacial dynamic friction and enhancing the wall slip. However, in contrast to the general alignment of the chain backbone in the flow direction, the short branches tend to be oriented more perpendicular (i.e., aligned in the neutral direction) than parallel to the backbone owing to their bonded and nonbonded intramolecular LJ interactions with the neighboring backbone atoms around the branch points. This orientational tendency of short-chain branches along the *z*-direction effectively increases the polymer–wall friction, diminishing the interfacial slip. These two contrasting contributions between the backbone and short branches cancel each other out to result in a nearly constant behavior of *d*_s_ in the weak flow regime for the SCB linear polymer (Figure 1). We further note that the short branches have intrinsically fast random Brownian kinetics owing to their very short characteristic relaxation time scale (e.g., ~0.06 ns for C_5_H_12_ at *T* = 450 K and *p* = 1 atm), and thus their dynamics are practically unaffected by the external flow field [25,27]. Such fast random movements of short branches along the chain backbone constantly disturb the overall chain conformation and tend to diminish the degree of structural deformation of the polymer chains in response to the applied flow. This further facilitates the overall chain dimension of SCB polymers in the neutral direction.

By combining the respective influences of the ring topology and short-chain branching on the interfacial dynamics, we can reliably predict the general behavior of *d*_s_ for the SCB ring polymer. As mentioned above, the ring polymer, owing to its closed-loop topology, has a larger chain dimension in the neutral direction in comparison to the linear analogue. Furthermore, the fast random motions of the highly mobile short branches along the backbone constantly disturb the overall chain conformation, leading to lesser degrees of chain stretch and orientation along the flow direction and also a relatively larger chain dimension along the neutral direction. Thus, both the ring geometry and short-chain branching are expected to diminish the overall chain extension and alignment in the flow direction and increase the chain dimension in the neutral direction, synergistically enhancing the dynamic friction of interfacial chains moving against the wall. We therefore consider that the SCB ring polymer would exhibit significantly lower values of $d_{s}$ in the weak flow regime in comparison to the other (linear, ring, and SCB linear) polymers, which is confirmed by the results shown in Figure 1.

Let us now examine the slip behavior of each polymer system in the intermediate flow regime. Two representative molecular mechanisms were identified for the interfacial linear polymer in this regime [23]: (i) an out-of-plane wagging mechanism (i.e., repetitive motions of the (outer) parts of the interfacial chains between detachment from the wall due to the external flow field and attachment to the wall due to the attractive polymer–wall interaction) and (ii) a disentanglement mechanism between the interfacial chains and nearby surrounding bulk chains via chain alignment and stretch along the flow direction. These two molecular processes effectively mitigate the movement of interfacial chains at the wall in the flow direction, resulting in an overall decreasing tendency of $d_{s}$ for the linear and ring polymers in the intermediate flow regime, as shown in Figure 1. Notably, interfacial ring polymers, in addition to the loop wagging mechanism, exhibit a loop migration dynamic mechanism where locally created loop segments can propagate along the chain toward the flow direction [26]. Because the loops can be created locally and randomly along the ring chain via thermal Brownian motion, the applied flow field, and intermolecular collisions, the loop wagging and loop migration dynamics may occur at any position of the chain, which is in contrast to the case of the linear polymer for which the out-of-plane wagging mechanism commonly occurs near the chain ends. Furthermore, the loop migration mechanism facilitates the movement of the whole ring chain in the flow direction and thus enhances the polymer slip at the wall. Additionally, the closed-loop topology of ring chains results in relatively fewer entanglement interactions compared to linear chains. These two factors together lead to a smaller decrease in $d_{s}$ for the ring polymer compared to the linear polymer in the intermediate flow regime (see Figure 1).

In contrast, both the SCB linear and SCB ring polymers exhibit nearly constant behavior for $d_{s}$ with respect to the shear rate throughout the weak and intermediate flow regimes. As mentioned previously, the highly mobile short branches make the overall chain structure more compact and less deformed (with highly curvy backbone structures) against the applied flow [25]. This structural feature entails smaller entanglement interactions between chains and less variation in the degree of entanglement with respect to the flow strength, ultimately weakening the degree of the out-of-plane wagging and disentanglement mechanisms and consequently affording the apparent constant behavior of $d_{s}$ for the SCB polymers.

In the strong flow regime, the external flow field is sufficiently strong to overcome the attractive polymer–wall interactions and cause intensive dynamical collisions between interfacial chains and the wall. This leads the interfacial chains to frequently detach from the wall and undergo irregular (chaotic) chain rotation and tumbling dynamics. This dynamic feature underlies a rapid increase in the degree of slip in this flow regime for all of the polymer systems (Figure 1).

It is interesting to note that whereas the tumbling dynamics occurs exclusively with the chain ends for the linear polymer, it can occur with any local loop(s) along the chain in the case of the ring polymer. This fact leads to two distinct types of tumbling mechanism: end-loop tumbling and center-loop tumbling [32]. The end-loop tumbling mechanism is induced by the local loops situated near the ends (outermost parts) of the stretched ring backbone along the flow direction, which is essentially similar to the typical end-over-end tumbling mechanism of the linear polymer. In contrast, the center-loop tumbling mechanism is driven by the loops located in the middle of the stretched ring backbone. Therefore, whereas end-loop tumbling practically occurs in the shear or flow-gradient (*xy*-)plane, the center-loop mechanism exhibits a diagonal chain rotation lying through both the *xy*- and *yz*-planes. These dynamic mechanisms were observed for both pure ring and SCB ring polymers at the interface under strong flow fields (specific quantification of the individual loop-tumbling mechanisms will be presented later).

Another interesting feature is that the interfacial SCB linear polymer exhibits not only the hairpin-like end-over-end tumbling behavior (similar to the linear polymer) but also additional distinct mechanisms such as head-roll and tail-roll tumbling dynamics, which is essentially caused by the short branches forming the compact head or tail parts along the chain backbone during the tumbling event [25]. However, such rolling mechanisms were rarely observed in the case of the SCB ring polymer studied here. This is attributed to the relatively larger chain dimension along the neutral direction for the SCB ring polymer compared to the corresponding SCB linear polymer in addition to the rather double-stranded stretched ring conformations at strong flow fields, both of which may have effectively impeded the whole chain rolling along the flow direction (additionally, the C_128_ backbone length of the present C_178_H_356_ SCB ring PE might not be sufficiently long to facilitate chain rolling). It is also important that the short branches promote the interfacial chain detachment from the wall by constantly disturbing the chain conformation via their fast random motions. This leads to a steeper increasing behavior of $d_{s}$ in the strong flow regime for both SCB linear and ring polymers in comparison to the corresponding pure linear and ring polymers.

**Figure 3:**
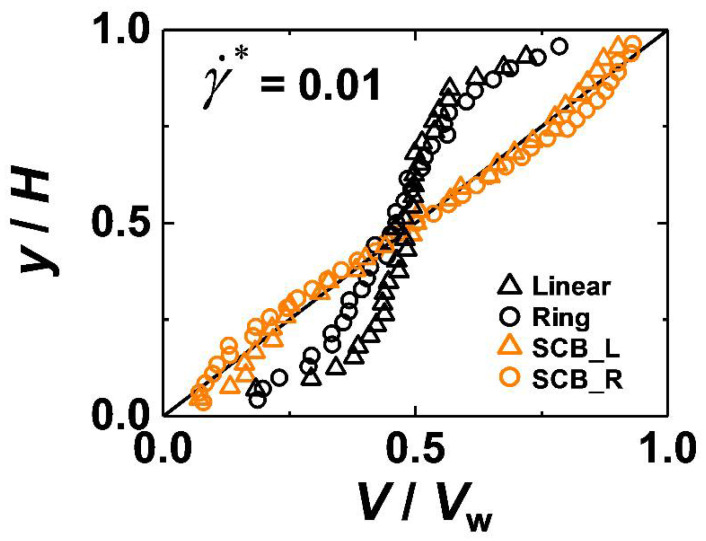
Streaming velocity profiles (normalized by the applied wall velocity $V_{w}$) along the velocity gradient direction at a certain intermediate shear rate for the simulated C_128_H_258_ linear (black triangles), C_128_H_256_ ring (black circles), C_178_H_358_ SCB linear (orange triangles), and C_178_H_356_ SCB ring (orange circles) PE melts. The solid line represents the ideal streaming velocity profile assuming the no-slip boundary condition.

Figure 3 displays the steady-state streaming velocity profiles in the flow direction along the velocity gradient direction for the four polymer systems at an intermediate flow strength. In general, the nonlinearity of the velocity profile for a polymeric material is associated with the degree of interfacial slip. As noted earlier, ${\dot{\gamma }}_{real}$ was calculated by applying linear regression to the streaming velocity data along the *y*-direction. Clearly, the pure linear and ring polymers exhibit a noticeable deviation from the ideal velocity profile (i.e., assuming the no-slip boundary condition) due to finite slip at the walls. Interestingly, the corresponding SCB linear and ring systems at the same shear rate display velocity profiles rather close to the ideal one ($d_{s}$ ≈ 0), indicating apparently small interfacial slip. This behavior results from the enhanced polymer–wall friction due to the highly mobile short branches. Furthermore, owing to the additional wall friction caused by its closed-loop geometry, the SCB ring polymer exhibits even smaller interfacial slip than the SCB linear polymer.

**Figure 4:**
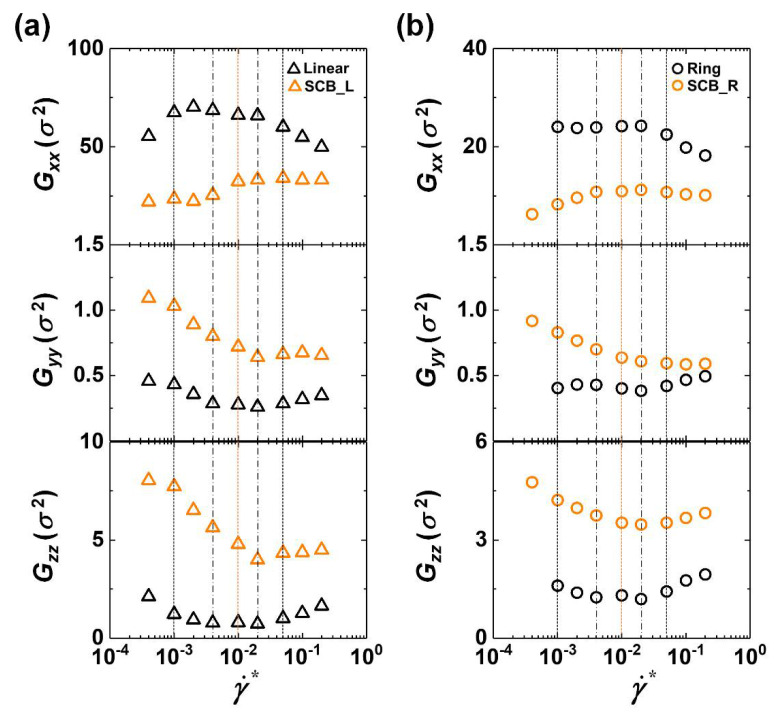
The *xx*-, *yy*-, and *zz*-components of the gyration tensor **G** ($G_{\alpha \beta }=\frac{1}{N}\sum_{i=1}^{N}\left(r_{i,\alpha }^{}-r_{c,\alpha }^{})(r_{i,\beta }^{}-r_{c,\beta }^{}\right)$, where $r_{i}^{}$ and $r_{c}^{}$ denote the position vectors of atom *i* and the chain center-of-mass with α, β = *x*, *y*, *z*) for the interfacial chains whose center-of-mass is located within a distance of 2.5 σ from the wall for the (**a**) C_128_H_258_ linear (black triangles) and C_178_H_358_ SCB linear (orange triangles) and (**b**) C_128_H_256_ ring (black circles) and C_178_H_356_ SCB ring (orange circles) systems as a function of the applied shear rate. To allow comparison with the pure ring and linear systems, only the chain backbone, excluding the short branches, was considered in the calculation of *G* for the SCB systems. The symbols and vertical lines have the same meaning as in Figure 1. The error bars are smaller than the size of the symbols unless otherwise indicated.

To further understand the interfacial characteristics in conjunction with the dynamic mechanisms, we analyzed the structural properties of the interfacial chains with centers of mass located within a distance of 2.5 σ from the wall. Figure 4 presents the *xx*-, *yy*-, and *zz*-components of the gyration tensor **G** of the interfacial chains for each system as a function of the applied flow strength. It should be noted that to examine the influence of short-chain branching on the overall chain structure, only the chain backbone (excluding the short branches) was included in the calculation of **G** for the SCB polymers.

First, as the shear rate increases in the weak flow regime, the linear polymer exhibits an increasing behavior of $G_{xx}$ (which approaches a plateau value) and a decreasing behavior of both $G_{zz}$ and $G_{yy}$. This is directly related to the *z*-to-*x* rotation mechanism of the interfacial chains with their decreased *y* dimension via chain alignment and stretch in the flow direction. Upon further increasing the flow strength to the intermediate flow regime, $G_{xx}$ shows almost constant behavior while both $G_{zz}$ and $G_{yy}$ continue to decrease until reaching a minimum value. These features can be understood by considering the aforementioned out-of-plane wagging and disentanglement mechanisms of the interfacial chains in the intermediate flow regime. Both mechanisms lead to significant suppression of the rotation and tumbling dynamics for the interfacial chains moving away from the wall. In the strong flow regime, as the shear rate increases, $G_{xx}$ appears to rapidly decrease, whereas both $G_{yy}$ and $G_{zz}$ display a gradual increase. This is directly associated with the irregular rotation and tumbling dynamic mechanism of the interfacial chains via their strong dynamical collisions with the wall, which facilitates the detachment of the interfacial chains from the wall and their frequent movement toward the bulk region. Overall similar behavior is exhibited by the ring polymer, which, however, owing to its intrinsic closed-loop molecular geometry, shows somewhat smaller variation in each component ($G_{xx}$, $G_{yy}$, and $G_{zz}$) with respect to the flow strength compared to the linear polymer. It is also noted that the ring topology gives rise to an increase in $G_{zz}$ and a decrease in $G_{yy}$.

In contrast, the SCB linear and ring polymers exhibit increasing behavior of $G_{xx}$ and decreasing behavior of $G_{yy}$ and $G_{zz}$ throughout the weak-to-intermediate flow regimes, with all components remaining almost constant in the strong flow regime. Furthermore, the SCB polymers display a significant reduction in *G_xx_* and increase in *G_yy_* and *G_zz_* in comparison to the corresponding pure linear and ring polymers. This feature is directly associated with the fact that the structural disturbance by the fast random motions of the short branches results in highly curvy backbone structures and lesser degrees of chain stretch and alignment to the flow direction for the SCB polymers.

It can be summarized that (i) the closed-loop ring geometry tends to reduce both $G_{xx}$ and $G_{yy}$ and promote $G_{zz}$ and (ii) short-chain branching tends to reduce $G_{xx}$ and promote both $G_{yy}$ and $G_{zz}$.

**Figure 5:**
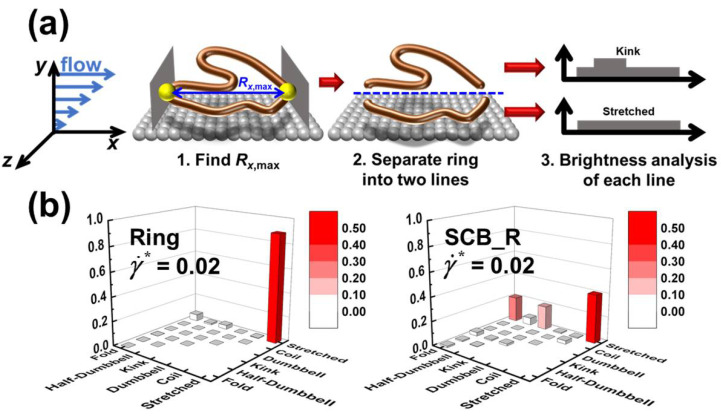
(**a**) Schematic depiction of the proposed Brightness analysis for a ring-shaped polymer chain. (**b**) Probability distribution functions (PDFs) resulting from the Brightness analysis applied to the interfacial chains for the pure ring (left panel) and SCB ring (right panel) polymers at a relatively high shear rate (${\dot{\gamma }}^{*}$ = 0.02). Note that the molecular configurations for each part of the ring backbone are categorized into six representative classes (stretched, coil, dumbbell, kink, half-dumbbell, and fold).

To investigate the distinctive structural characteristics of the interfacial SCB ring polymer, we conducted the Brightness analysis to categorize the mesoscale chain structures into several representative configurational classes (namely, stretched, coil, dumbbell, kink, half-dumbbell, and fold) on the basis of the monomer distribution along the chain [40,41]. For this analysis, we devised a new scheme that provides detailed structural information for interfacial ring-shaped polymer chains. Specifically, (i) we first identify the longest dimension ($R_{x,max}$) of the ring backbone in the flow direction, (ii) we then divide the ring backbone into two linear parts separated by the cutting line of $R_{x,max}$, and (iii) we finally perform the Brightness analysis for each part separately and categorize the respective molecular configurations into the six representative classes, as depicted in Figure 5a.

Figure 5b presents the probability distributions for the combined configurational classes of the individual parts of the interfacial chains for the simulated ring and SCB ring systems at a relatively high shear rate (${\dot{\gamma }}^{*}$ = 0.02). It can be seen that the pure ring system exhibits mostly the “stretched–stretched” configuration for the two parts, indicating well-aligned and extended double-stranded ring conformations. In contrast, the SCB ring system displays fairly large proportions of the “fold–stretched” and “kink–stretched” configurations, in addition to the predominant “stretched–stretched” configuration. These structural features are closely associated with the overall curvy extended chain conformations of the interfacial SCB ring chains caused by the disturbance exerted by the highly mobile short branches along the ring backbone. The Brightness results can be seen to be consistent with the results for the gyration tensor *G* shown in Figure 4.

**Figure 6:**
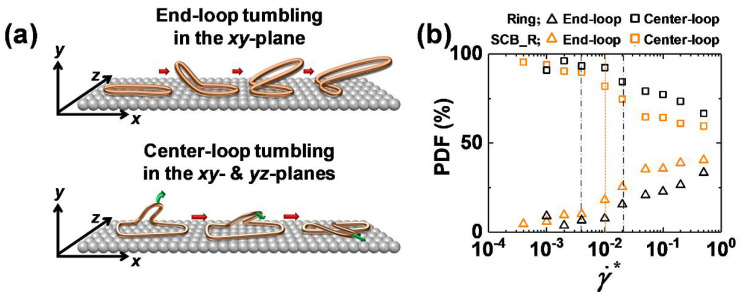
(**a**) Schematic depiction of the two types of loop tumbling mechanisms (end-loop tumbling and center-loop tumbling) of the interfacial ring chains. (**b**) Proportion of end-loop tumbling (triangles) and center-loop tumbling (squares) of the interfacial chains as a function of the shear rate for the pure ring (black symbols) and SCB ring (orange symbols) polymers. The vertical lines have the same meaning as in Figure 1.

As mentioned earlier, the ring polymer exhibits two types of tumbling dynamics at high flow fields: end-loop and center-loop tumbling (Figure 6a). Both mechanisms are induced by the local loop(s) randomly created along the closed ring backbone via thermal Brownian motion, the applied flow field, and intermolecular collisions between chains [32]. Here we further quantified the proportion of each tumbling mechanism for the interfacial ring and SCB ring polymers as a function of the shear rate (Figure 6b). We first noticed that the center-loop tumbling is generally more dominant than the end-loop tumbling for both pure ring and SCB ring polymers. This can be understood by considering that, whereas the center-loop tumbling can be induced by any local loop along the stretched ring backbone, the end-loop tumbling is induced only by local loops located near the ends of the stretched ring backbone. Notably, the proportion of center-loop tumbling appears to be smaller for the SCB ring polymer than for the pure ring polymer. This is attributed to the relatively larger *z*-dimension of the interfacial SCB ring chains compared to the interfacial ring chains due to the short branches in the former (Figure 4).

## 4. Conclusions

In this study, we performed a comprehensive analysis of the interfacial structural and dynamical behavior of confined polymer melt systems possessing various molecular architectures (linear, ring, SCB linear, and SCB ring) under shear flow using atomistic NEMD simulations. We placed a particular focus on the combined rheological influence of the closed-loop ring geometry and short-chain branching on the general structure and dynamics of the interfacial chains. In doing so, we examined the degree of interfacial slip, the underlying characteristic molecular mechanisms, and the detailed chain conformations for interfacial chains with respect to their molecular architectures.

In general, the interfacial linear and ring polymers exhibit three distinct characteristic regimes for the degree of slip ($d_{s}$) with respect to the applied shear rate: an increasing, decreasing, and increasing behavior of $d_{s}$ in the weak, intermediate, and strong flow regimes, respectively. In contrast, the interfacial SCB linear and SCB ring polymers display almost constant behavior of $d_{s}$ throughout the weak and intermediate flow regimes followed by rapidly increasing behavior of $d_{s}$ in the strong flow regime.

To elucidate the interfacial slip behavior, it is very informative to analyze the fundamental molecular mechanisms with respect to the three representative (weak, intermediate, and strong) flow regimes. In the weak flow regime, all of the interfacial polymers (linear, ring, SCB linear, and SCB ring) undergo *z*-to-*x* chain rotation from the neutral direction to the flow direction, which effectively reduces the dynamic wall friction against chain movement along the flow direction. However, in comparison with its linear analogue, the ring polymer possesses a relatively longer chain dimension in the neutral direction owing to its intrinsic closed-loop geometry, which enhances the dynamic friction of the interfacial ring chains moving against the wall in the flow direction. In the case of the interfacial SCB polymers, whereas the main chain backbone becomes gradually aligned to the flow direction with increasing flow strength (thus decreasing the dynamic wall friction), the short branches tend to be oriented more perpendicular (i.e., aligned in the neutral direction) than parallel to the backbone (thus increasing the dynamic wall friction) in conjunction with their intrinsically fast random motions that are practically unaffected by the imposed flow fields. These two contrasting factors cancel each other out to result in almost constant $d_{s}$ in the weak flow regime for the interfacial SCB polymers. Furthermore, owing to its relatively larger chain dimension in the neutral direction associated with the ring topology, the SCB ring polymer exhibits the lowest degree of slip among all of the polymers.

The basic molecular mechanisms in the intermediate flow regime are out-of-plane chain wagging (i.e., repetitive motions between chain detachment from and chain attachment to the wall) and disentanglement of the interfacial chains from the surrounding bulk chains. The interfacial ring chains exhibit distinct dynamics referred to as loop migration (i.e., the local loops propagate along the chain backbone toward the flow direction) as well as the loop wagging mechanism. In comparison, the SCB polymers exhibit overall weaker out-of-plane wagging and disentanglement mechanisms owing to their more compact and less deformed (highly curvy) chain structures caused by the fast random movement of short branches along the backbone. Again, the interfacial SCB ring polymer displays the lowest value of $d_{s}$ throughout the weak and intermediate flow regimes as a consequence of the synergetic effects of the ring topology and short-chain branching.

In the strong flow regime, the flow field is sufficiently intense to overcome the attractive polymer–wall interactions and induce frequent interfacial chain detachment from the wall and irregular (chaotic) chain rotation and tumbling dynamics via strong dynamical collisions between the interfacial chains and the wall. This induces a rapid increase in the interfacial slip in this flow regime for all of the polymer systems, irrespective of the molecular architecture. In addition, owing to the enhanced interfacial chain detachment from the wall via the constant structural disturbance exerted by the highly mobile short branches, the SCB polymers exhibit even steeper increasing $d_{s}$ behavior in the strong flow regime in comparison to the corresponding pure linear and ring polymers.

Another interesting feature is that the interfacial SCB linear chains exhibit not only hairpin-like tumbling behavior but also two types of rolling dynamics (head-roll and tail-roll tumbling mechanisms) that are essentially induced by the short branches. However, such rolling dynamics were rather absent from the SCB ring system studied here. This is attributed to the difficulties in chain rolling for the stretched double-stranded rings with a relatively large chain dimension along the neutral direction.

It is also notable that the ring-shaped polymers essentially exhibit two types of loop tumbling mechanisms (end-loop and center-loop tumbling) at strong flow fields. The end-loop and center-loop tumbling mechanisms are induced by local loops situated near the ends and in the middle of the stretched ring backbone along the flow direction, respectively. As such, while the end-loop tumbling occurs mostly in the *xy*-plane, the center-loop tumbling occurs diagonally through both the *xy*- and *yz*-planes. It is found that the interfacial SCB ring polymer, in association with its larger chain dimension in the neutral direction via short-chain branching, exhibits a relatively smaller proportion of center-loop tumbling in comparison with the pure ring polymer.

In respect to the overall chain dimension, the closed-loop ring topology tends to enhance $G_{zz}$ but diminish both $G_{xx}$ and $G_{yy}$, while short-chain branching tends to enhance both $G_{yy}$ and $G_{zz}$ but diminish $G_{xx}$.

## Supplementary Materials

The following is available online at https://www.mdpi.com/2073-4360/12/12/3068/s1: the simulation methodology.

## References

[1] Stevens M.J., Mondello M., Grest G.S., Cui S.T., Cochran H.D., Cummings P.T. (1997). Comparison of shear flow of hexadecane in a confined geometry and in bulk. J. Chem. Phys..

[2] Baschnagel J., Vamik F. (2005). Computer simulations of supercooled polymer melts in the bulk and in confined geometry. J. Phys. Condens. Matter.

[3] Gee M.L., McGuiggan P.M., Israelachvili J.N., Homola A.M. (1990). Liquid to solidlike transitions of molecularly thin films under shear. J. Chem. Phys..

[4] Van Alsten J., Granick S. (1988). Molecular tribometry of ultrathin liquid films. Phys. Rev. Lett..

[5] Christenson H.K., Gruen D.W.R., Horn R.G., Israelachvili J.N. (1987). Structuring in liquid alkanes between solid surfaces: Force measurements and mean-field theory. J. Chem. Phys..

[6] Tordella J.P. (1957). Capillary flow of molten polyethylene-A photographic study of melt fracture. Trans. Soc. Rheol..

[7] Kalika D.S., Denn M.M. (1987). Wall slip and extrudate distortion in linear low-density polyethylene. J. Rheol..

[8] Denn M.M. (2001). Extrusion instabilities and wall slip. Annu. Rev. Fluid Mech..

[9] Jabbarzadeh A., Atkinson J.D., Tanner R.I. (2000). Effect of the wall roughness on slip and rheological properties of hexadecane in molecular dynamics simulation of Couette shear flow between two sinusoidal walls. Phys. Rev. E.

[10] Priezjev N.V. (2007). Effect of surface roughness on rate-dependent slip in simple fluids. J. Chem. Phys..

[11] Lee T., Charrault E., Neto C. (2014). Interfacial slip on rough, patterned and soft surfaces: A review of experiments and simulations. Adv. Colloid Interface Sci..

[12] Nagayama G., Cheng P. (2004). Effects of interface wettability on microscale flow by molecular dynamics simulation. Int. J. Heat Mass Transf..

[13] Priezjev N.V., Darhuber A.A., Troian S.M. (2005). Slip behavior in liquid films on surfaces of patterned wettability: Comparison between continuum and molecular dynamics simulations. Phys. Rev. E.

[14] Dijkstra M. (1997). Confined thin films of linear and branched alkanes. J. Chem. Phys..

[15] Gupta S.A., Cochran H.D., Cummings P.T. (1997). Shear behavior of squalane and tetracosane under extreme confinement. I. Model, Simulation method, and interfacial slip. J. Chem. Phys..

[16] Ansari M., Inn Y.W., Sukhadia A.M., DesLauriers P.J., Hatzikiriakos S.G. (2013). Wall slip of HDPEs: Molecular weight and molecular weight distribution effects. J. Rheol..

[17] Sabzevari S.M., Cohen I., Wood-Adams P.M. (2014). Wall slip of tridisperse polymer melts and the effect of unentangled versus weakly entangled chains. Macromolecules.

[18] Gao J., Luedtke W.D., Landman U. (1997). Layering transitions and dynamics of confined liquid films. Phys. Rev. Lett..

[19] Cui S.T., Cummings P.T., Cochran H.D. (2001). Molecular simulation of the transition from liquidlike to solidlike behavior in complex fluids confined to nanoscale gaps. J. Chem. Phys..

[20] Mooney M. (1931). Explicit formulas for slip and fluidity. J. Rheol..

[21] Khare R., de Pablo J.J., Yethiraj A. (1996). Rheology of confined polymer melts. Macromolecules.

[22] Hénot M., Drockenmuller E., Léger L., Restagno F. (2018). Sensing adsorption kinetics through slip velocity measurements of polymer melts. Euro. Phys. J. E.

[23] Jeong S., Cho S., Kim J.M., Baig C. (2017). Molecular mechanisms of interfacial slip for polymer melts under shear flow. J. Rheol..

[24] Cho S., Jeong S., Kim J.M., Baig C. (2017). Molecular dynamics for linear polymer melts in bulk and confined systems under shear flow. Sci. Rep..

[25] Jeong S., Kim J.M., Cho S., Baig C. (2017). Effect of short-chain branching on interfacial polymer structure and dynamics under shear flow. Soft Matter.

[26] Jeong S., Cho S., Kim J.M., Baig C. (2018). Interfacial molecular structure and dynamics of confined ring polymer melts under shear flow. Macromolecules.

[27] Kim J.M., Baig C. (2016). Communication: Role of short chain branching in polymer structure and dynamics. J. Chem. Phys..

[28] Jeong S.H., Kim J.M., Baig C. (2017). Rheological influence of short-chain branching for polymeric materials under shear with variable branch density and branching architecture. Macromolecules.

[29] Yoon J., Kim J., Baig C. (2016). Nonequilibrium molecular dynamics study of ring polymer melts under shear and elongation flows: A comparison with their linear analogs. J. Rheol..

[30] Li Y., Hsiao K.-W., Brockman C.A., Yates D.Y., Robertson-Anderson R.M., Kornfield J.A., San Francisco M.J., Schroeder C.M., Mckenna G.B. (2015). When end meet: Circular DNA stretches differently in elongational flows. Macromolecules.

[31] Yong C.D., Qian J.R., Marvin M., Sing C.E. (2019). Ring polymer dynamics and tumbling-stretch transitions in planar mixed flows. Phys. Rev. E.

[32] Jeong S.H., Cho S., Roh E.J., Ha T.Y., Kim J.M., Baig C. (2020). Intrinsic surface characteristics and dynamic mechanisms of ring polymers in solution and melt under shear flow. Macromolecules.

[33] Baig C., Edwards B.J., Keffer D.J., Cochran H.D. (2005). A proper approach for nonequilibrium molecular dynamics simulations of planar elongational flow. J. Chem. Phys..

[34] Baig C., Edwards B.J., Keffer D.J., Cochran H.D. (2005). Rheological and structural studies of liquid decane, hexadecane, and tetracosane under planar elongational flow using nonequilibrium molecular-dynamics simulations. J. Chem. Phys..

[35] Nosé S. (1984). A molecular dynamics method for simulations in the canonical ensemble. Mol. Phys..

[36] Hoover W.G. (1985). Canonical dynamics: Equilibrium phase-space distributions. Phys. Rev. A.

[37] Martin M.G., Siepmann J.I. (1999). Novel configurational-bias Monte Carlo method for branched molecules. Transferable potentials for phase equilibria. 2. United-atom description of branched alkanes. J. Phys. Chem. B.

[38] Tuckerman M., Berne B.J., Martyna G.J. (1992). Reversible multiple time scale molecular dynamics. J. Chem. Phys..

[39] Baig C., Mavrantzas V.G., Kröger M. (2010). Flow effects on melt structure and entanglement network of linear polymers: Results from a nonequilibrium molecular dynamics simulation study of a polyethylene melt in steady shear. Macromolecules.

[40] Venkataramani V., Sureshkumar R., Khomami B. (2008). Coarse-grained modeling of macromolecular solutions using a configuration-based approach. J. Rheol..

[41] Kim J.M., Edwards B.J., Keffer D.J., Khomami B. (2010). Dynamics of individual molecules of linear polyethylene liquids under shear: Atomistic simulation and comparison with a free-draining bead-rod chain. J. Rheol..

