# Supplementary material for: Structural and Dynamical Characteristics of Short-Chain Branched Ring Polymer Melts at Interface under Shear Flow
Source: Polymers (Basel). 2020 Dec 21;12(12):3068. doi: 10.3390/polym12123068 (PMC7767370; doi:10.3390/polym12123068)
Supplement: Supplementary file 1 [file polymers-12-03068-s001.pdf]

Structural and Dynamical Characteristics of Short-Chain Branched Ring Polymer Melts at Interface under Shear Flow

Seung Heum Jeong†, Soowon Cho†, Tae Yong Ha, Eun Jung Roh, and Chunggi Baig*

Simulation Methodology

The well-known Transferable Potentials for Phase Equilibria (TraPPE) united-atom model was adopted in the simulations [1]. In the TraPPE model, nonbonded intra- and intermolecular interaction were modeled by a pairwise 6-12 Lennard-Jones (LJ) potential:

$$U_{LJ}(r) = 4\varepsilon_{ij}\left[\left(\frac{\sigma_{ij}}{r}\right)^{12} - \left(\frac{\sigma_{ij}}{r}\right)^{6}\right] \tag{1}$$

where ε/k_B for the CH, CH_2, and CH_3 are 10 K, 46 K, and 98K, respectively, and σ for the CH, CH_2, and CH_3 are 4.68 Å, 3.95 Å, and 3.75 Å, respectively, where k_B denotes the Boltzmann constant. The cut-off distance for both atom-atom and atom-wall is 2.5 σ_{ij}. The three bonded (bond-stretching, bond-bending, and bond-torsional) interactions are modeled by the following potentials:

$$U_{stretching}(l) = \frac{k_{str}}{2}(l - l_{eq})^2 \tag{2}$$

$$U_{bending}(\theta) = \frac{k_{ben}}{2}(\theta - \theta_{eq})^2 \tag{3}$$

$$U_{torsional}(\phi) = \sum_{m=0}^{3} a_m \cos^m \phi \tag{4}$$

where the bond-stretching constant is k_{str}/k_B = 452,900 K/ Å and the equilibrium bond length l_{eq} = 1.54 Å. The bond-bending parameter k_θ is equal to 62.500 K/rad^2 and the equilibrium bending angle θ_{eq} is 114°, 112°, and 109.47° for CH_x-CH_2-CH_y, CH_x-CH-CH_y, and CH_x-C-CH_y, respectively, where x and y are equal to 2 or 3. The bond-torsional parameters are such that (i) a_0 = 1010 K, a_1 = 2019 K, a_2 = 136.4, and a_3 = –3165K for CH_x-CH_2-CH_2-CH_y, (ii) a_0 = 395.2 K, a_1 = 895.1 K, a_2 = 223.7 K, and a_3 = –1765.2 K for CH_x-CH_2-CH-CH_y, and (iii) a_0 = 461.3 K, a_1 = 1384.1 K, a_2 = 0 K, and a_4 = –1845.2 K for CH_x-CH_2-CH_2-CH_y [note that here $\phi = 0$ indicates the (most stable) *trans*-conformation, whereas $\phi = 180°$ refers to the *cis*-conformation].

The simulation systems were confined by rigid simple cubic lattice walls where the lattice parameter of the simple cubic wall was set equal to σ_w = 1.33 σ_f where σ_f is σ_{CH_2} [2]. The LJ energy parameter of wall atoms in all PE systems was set as ε_w/k_B = 939 K, which is comparable to that of a mica surface (~200-400 mJ/m^2) [3]. The simulation systems were confined by rigid simple cubic lattice walls composed of 676, 1352, and 2028 atoms for each of the $C_{128}H_{258}$ linear, $C_{128}H_{256}$ ring, and $C_{178}H_{358}$ short-chain branched (SCB) linear PE systems, and 1224, 2448, and 3672 atoms for the $C_{178}H_{356}$ SCB ring PE system. Each wall atom was kept fixed in its lattice site during the simulations. The interaction parameters between wall atom w and polymer atom f were specified as $\varepsilon_{wf}=(\varepsilon_w\varepsilon_f)^{1/2}$ and $\sigma_{wf}=(\sigma_w+\sigma_f)/2$. The cut-off distance of the LJ polymer-wall interaction was set as $2.5\sigma_{wf}$. Therefore, the polymer-wall interaction can be active up to two layers at most. As such, each top and bottom wall boundary was set by two layered simple cubic lattice walls for all PE confined systems in this study.

The atomistic nonequilibrium molecular dynamics (NEMD) simulations were executed with the p-SLLOD algorithm with a Nosé-Hoover thermostat [4-7]:

$$
\begin{aligned}
\dot{\mathbf{r}}_i &= \frac{\mathbf{p}_i}{m_i} + \mathbf{r}_i \cdot \nabla\mathbf{u} \\
\dot{\mathbf{p}}_i &= \mathbf{F}_i(\mathbf{r}) - \mathbf{p}_i \cdot \nabla\mathbf{u} - m_i \mathbf{r}_i \cdot \nabla\mathbf{u} \cdot \nabla\mathbf{u} - \frac{p_s}{Q}\mathbf{p}_i - \frac{p_s}{Q}(m_i \mathbf{r}_i \cdot \nabla\mathbf{u} - m_i \mathbf{U}(\mathbf{r}_i)) \\
\dot{s} &= \frac{p_s}{Q} \\
\dot{p}_s &= F_s(\mathbf{p}_i)
\end{aligned}
\tag{5}
$$

where $\mathbf{r}_i$, $\mathbf{p}_i$, and $\mathbf{F}_i$ are the position, (nominal) peculiar momentum, and force vector of particle i of mass m_i. s and p_s are coordinate-like and momentum-like variables, respectively, of the Nosé-Hoover thermostat. $Q = DNk_BT\tau^2$ is thermostat mass parameter, where D and τ are the dimensionality and relaxation time parameter, respectively. The τ was set equal to 0.24 ps for all simulations. $\mathbf{U}(\mathbf{r}_i)$ is the streaming velocity at position $\mathbf{r}_i$, which was evaluated based on a 5th order polynomial fitting throughout the total region in every MD step during simulation [2]. The real peculiar momentum $\mathbf{p}_i^{\text{real}}$ of each atom was then calculated by subtracting the streaming velocity at its position from its laboratory momentum:

$$
\dot{\mathbf{p}}_i^{\text{real}} = \mathbf{p}_i + m_i \mathbf{r}_i \cdot \nabla\mathbf{u} - m_i \mathbf{U}(\mathbf{r}_i) \tag{6}
$$

The $\nabla\mathbf{u}$, homogeneous velocity gradient tensor, is expressed as

$$
\nabla\mathbf{u} = \begin{bmatrix} 0 & 0 & 0 \\ \dot{\gamma} & 0 & 0 \\ 0 & 0 & 0 \end{bmatrix} \tag{7}
$$

where $\dot{\gamma}$ is the shear rate.

The bulk polymer configurations were initially constructed via an efficient Monte Carlo algorithm in the Material Studio (Accelrys Inc.) software package subsequently subjected to the energy minimization and pre-equilibration procedures. Then, simple cubic lattice walls were placed at the top and bottom boundary of the system in the confining (y-)direction to accommodate the initial bulk polymer system. We then applied isothermal-isobaric (*NPT*) molecular dynamics simulations to the confined system for a sufficiently long time to attain the equilibrium density of the system at P = 1atm and T = 450 K.

References

1. Martin, M. G.; Siepmann, J. I. Novel configurational-bias Monte Carlo method for branched molecules. Transferable potentials for phase equilibria. 2. United-atom description of branched alkanes. *J. Phys. Chem. B* **1999**, *103*, 4508-4517.
2. Jeong, S.; Cho, S.; Kim, J. M.; Baig, C. Molecular mechanisms of interfacial slip for polymer melts under shear flow. *J. Rheol.* **2017**, *61*, 253-264.
3. Cui, S. T.; Cummings, P. T.; Cochran, H. D. Molecular simulation of the transition from liquidlike to solidlike behavior in complex fluids confined to nanoscale gaps. *J. Chem. Phys.* **2001**, *114*, 7189-7195.
4. Nosé, S. A molecular dynamic method for simulations in the canonical ensemble. *Mol. Phys.* **1984**, *52*, 255-268.
5. Hoover, W. G. Canonical dynamics: Equilibrium phase-space distributions. *Phys. Rev. A* **1985**, *31*, 1695-1697.
6. Baig, C.; Edwards, B. J.; Keffer, D. J.; Cochran, H. D. A proper approach for nonequilibrium molecular dynamics simulations of planar elongational flow. *J. Chem. Phys.* **2005**, *122*, 114103.
7. Baig, C.; Edwards, B. J.; Keffer, D. J.; Cochran, H. D. Rheological and structural studies of liquid decane, hexadecane, and tetracosane under planar elongational flow using nonequilibrium molecular-dynamics simulations. *J. Chem. Phys.* **2005**, *122*, 184906.
